# Anticandidal Activity of In Situ Methionine γ-Lyase-Based Thiosulfinate Generation System vs. Synthetic Thiosulfinates

**DOI:** 10.3390/ph16121695

**Published:** 2023-12-07

**Authors:** Svetlana Revtovich, Anna Lyfenko, Yaroslav Tkachev, Vitalia Kulikova, Vasiliy Koval, Vladimir Puchkov, Natalya Anufrieva, Pavel Solyev, Elena Morozova

**Affiliations:** Engelhardt Institute of Molecular Biology of the Russian Academy of Sciences, 119991 Moscow, Russia; svetla21@mail.ru (S.R.); lyfenkoanna@yandex.ru (A.L.); yaroslav@eimb.ru (Y.T.); vitviku@yandex.ru (V.K.); tokojami@yandex.ru (V.K.); noldor.9@ya.ru (V.P.); nvanufrieva@rambler.ru (N.A.)

**Keywords:** methionine γ-lyase-based thiosulfinates generation system, ^1^H NMR kinetic study, anticandidal activity, antimycotics synergy

## Abstract

*Candida albicans* and non-albicans *Candida* species are a common cause of human mucosal infections, as well as bloodstream infections and deep mycoses. The emergence of resistance of *Candida* spp. to antifungal drugs used in practice requires the search for new antimycotics. The present study unravels the antifungal potential of the synthetic dialk(en)ylthiosulfinates in comparison with an enzymatic in situ methionine γ-lyase-based thiosulfinate generation system (TGS). The kinetics of the TGS reaction, namely, the methionine γ-lyase-catalyzed β-elimination of S-alk(en)yl-L-cysteine sulfoxides, was investigated via ^1^H NMR spectroscopy for the first time, revealing fast conversion rates and the efficient production of anticandidal dialk(en)ylthiosulfinates. The anticandidal potential of this system vs. synthetic thiosulfinates was investigated through an in vitro assay. TGS proved to be more effective (MIC range 0.36–1.1 μg/mL) than individual substances (MIC range 0.69–3.31 μg/mL). The tested preparations had an additive effect with the commercial antimycotics fluconazole, amphotericin B and 5-flucytosine demonstrating a fractional inhibitory coefficient index in the range of 0.5–2 μg/mL. TGS can be regarded as an attractive candidate for the targeted delivery of antimycotic thiosulfinates and for further implementation onto medically implanted devices.

## 1. Introduction

Yeast-like fungi of the genus *Candida*, along with bacteria, such as *Pseudomonas aeruginosa*, *Escherichia coli* and *Staphylococcus aureus*, are the most common sources of nosocomial infections. Commonly, *Candida albicans* is a harmless human commensal, but in certain conditions, it can cause mucosal infections in healthy people, sometimes skin or nail infections, while immunocompromised patients suffer from severe systemic and often fatal bloodstream infections caused by this pathogen [1]. The ability of *C. albicans* to form biofilms on the surfaces of tissues and implanted medical devices increases the risk of the microorganism resistance to commercial antifungal drugs and leads to increased therapy costs and negative outcomes of diseases [2,3]. The current arsenal of antifungal drugs is limited; many of them exhibit high toxicity during long-term treatment and thus necessitate the search for new antifungal compounds. Treatment of the implants with biodegradable low-toxic prodrugs and the implication of surface-applied enzyme systems could fix this issue. It should be noted that the latest antifungal agents used in medical practice are compounds of natural origins, such as echinocandins and sordarins [4]. Medical plants have long been a perspective source for natural compounds with antibacterial and antifungal properties; one of the most prominent examples from ancient times is garlic. In recent times, it was shown that diallylthiosulfinate (allicin), formed in the plants of the *Allium* genus as a result of S-allyl-L-cysteine sulfoxide (alliin) degradation under the action of pyridoxal-5′-phosphate-dependent enzyme alliinase (EC 4.4.1.4), possesses a broad range of biological activities, including antifungal [5,6]. The fungicidal effect of garlic extract and allicin against the pathogenic fungi *Aspergillus, Candida, Cryptococcus*, *Trichophyton*, *Trichosporon*, *Rhodotorula* and other species is well established [7,8]. But despite the promising antimicrobial properties of allicin, its usage is limited by its instability at room temperature and a short half-life in the bloodstream [5]. Alkylthiosulfinates present in garlic extract in minor amounts are more stable and may be considered potential antimicrobial agents; having the same mode of action as allicin, they demonstrate a comparable antibacterial efficacy against some Gram-positive and Gram-negative bacteria [9]. In addition, thiosulfinates are volatile and can be used against pulmonary infections. However, allicin and, with a high probability, its analogues are also toxic to humans [10,11]. To overcome this issue, a new method of antifungal therapy against *Aspergillus fumigates* using the system of the targeted production of allicin on the surface of a pathogenic cell was proposed [12]. Briefly, the conjugate of alliinase with a monoclonal antibody was bound to conidia and hyphae of *A. fumigates*, and in the presence of alliin, it produced allicin, which caused the death of the pathogen without affecting mouse lung epithelial cells [12]. The work constituted the first example of an antibody-directed enzyme prodrug therapy candidate for antimicrobial treatment. However, further studies have not been developed, possibly due to the fact that recombinant alliinase has not been obtained yet.

**Figure 1:**
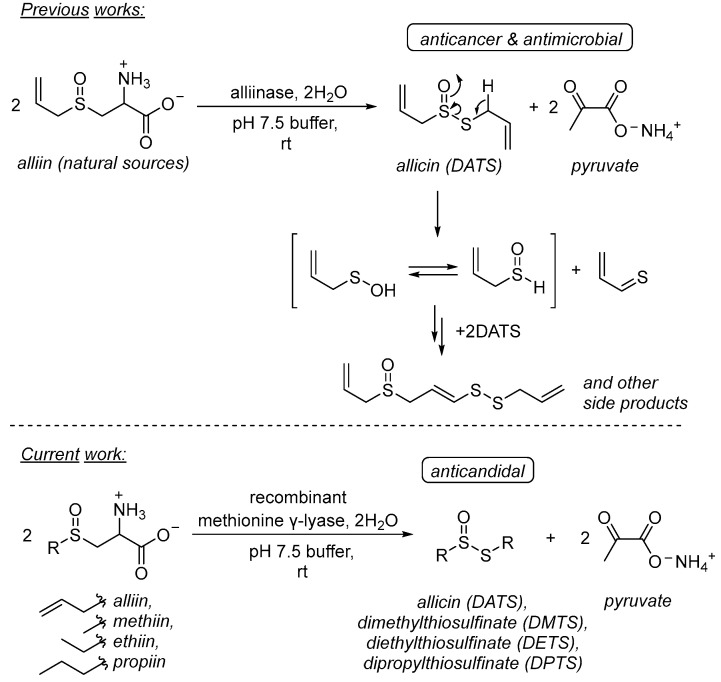
Thiosulfinate generation system (TGS) based on the alliinase- and methionine γ-lyase-catalyzed β-elimination of S-alk(en)yl-L-cysteine sulfoxides.

Instead, to succeed in the production of the enzyme prodrug therapy for anticandidal therapeutic purposes, we propose to use another closely related to alliinase enzyme (Figure 1).

Recently, we have shown that a binary system of methionine γ-lyase (EC 4.4.1.11, MGL)/S-alk(en)yl-L-cysteine sulfoxides possesses antimicrobial activity in vitro and in vivo [13,14]. This system generates antipathogenic compounds both from natural substrates and from their synthetic analogues, and the core enzyme can maintain its activity at the targeted surface via encapsulation. These studies have been investigated for the treatment of bacterial infections in vitro and in vivo, as well as applied for targeted delivery to breast cancer cells [14,15]. The second components of the binary system—S-alk(en)yl-L-cysteine sulfoxides—are found only in plants and can be chemically synthesized from readily available reagents in two steps with excellent yields and remain intact without the influence of external factors. Despite the feasibility of the recombinant production of MGL, its implication as an antifungal drug candidate has never been studied previously.

In our study, we evaluate the antifungal activity of the synthetic dialk(en)ylthiosulfinates in comparison with thiosulfinates generated in situ using a thiosulfinate-generation system (TGS), based on the binary system MGL/S-alk(en)yl-L-cysteine sulfoxides against the growth of *C. albicans*. The antimicrobial interactions of all tested preparations in combinations with commercial membrane- and DNA-targeting antifungals were classified via a checkerboard assay. The kinetic aspects of thiosulfinate formation using the TGS have been investigated.

To our knowledge, the investigation of the antifungal and kinetic properties of the TGS in comparison with synthetic analogues is performed for the first time.

## 2. Results and Discussion

### 2.1. ^1^H NMR Kinetic Study of β-Elimination Reaction of S-alk(en)yl-L-Cysteine Sulfoxides Catalyzed by C115HMGL

MGL-catalyzed thiosulfinate production has been known for several years; nevertheless, its kinetics has never been studied using NMR, as well as product consistency based on the theoretical mechanism involved. NMR monitoring of the enzymatic conversion of S-alk(en)yl-L-cysteine sulfoxides showed that products of the reaction were consistent with the known mechanism [16]. An increasing signal at 2.33 ppm indicated the formation of pyruvate over the course of the reaction. This signal, along with two characteristic signals of thiosulfinates, corresponding to the terminal protons of alk(en)yl substituents, were monitored. Their assignment to the intermediate sulfenic acid or thiol moieties could not be precisely established. Herein, to differentiate their nature we call them “low field” (LF) and “high field” (HF) signals.

^1^H NMR signal chemical shifts and multiplicities in the spectra of the enzymatic reaction mixture were fairly consistent (within ±0.005 ppm) with the ones obtained for synthetic thiosulfinates in aqueous solution, taking into consideration an overlap with the substrate signals. A corresponding spectroscopic data summary is given in Table 1. Signal shapes of CH_2_ and CH protons in DPTS and DATS are labeled as multiplets due to their complexity. No noticeable amounts of thiosulfinate degradation products were revealed via ^1^H NMR in enzymatic reaction mixtures.

**Figure 2:**
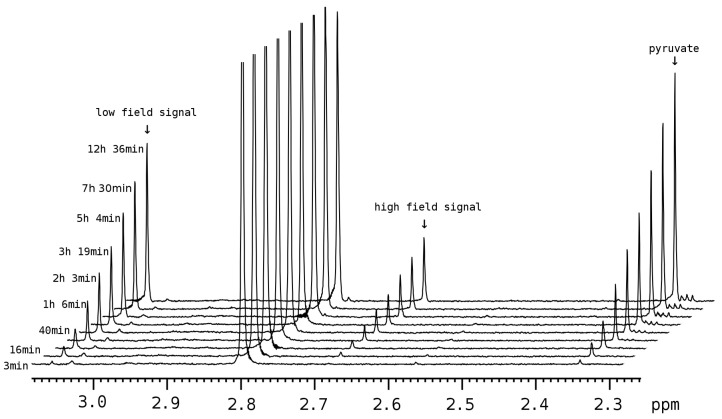
Evolution of the monitored ^1^H NMR signals during the production of DMTS by MGL. The signal at 2.8 ppm belongs to the substrate.

The least structurally encumbered DMTS demonstrates the evolution of its signals over the course of the reaction (Figure 2).

Kinetic data plots are given in the Supplementary material file. For each of the four products, strict proportionality was found between the intensities of all these signals. Therefore, they all reflect the same plot in kinetics. An analysis of the obtained kinetic data showed a significant slowdown in the reaction rate as compared to the Michaelis–Menthen model of an enzymatic reaction. It was expected, since thiosulfinate products are reactive towards thiol groups of the enzyme (particularly, Cys245 of C115H MGL) [17]. However, these data could not be meaningfully fit into a simple model of the Michaelis–Menthen mechanism with additional inactivation of the enzyme mediated by the produced thiosulfinate. A more complex, potentially multi-stage mechanism of the enzyme inactivation mediated by the product can be suggested. Due to this complication, we have calculated estimate values for initial turnover rates and slowdown rate half-conversion times. The resulting kinetic parameters are given in Table 2.

The conversion efficiency, both maximizing the initial turnover rate and minimizing the slowdown rate, decreased in the order DATS > DPTS ≈ DETS > DMTS. The latter was found to be profoundly inefficient compared to other thiosulfinates, while the kinetics of DPTS and DETS production were quite similar. DATS was found to be the most efficiently produced from a substrate series.

### 2.2. Antifungal Activity of Synthetic Dialk(en)ylthiosulfinates and TGS against C. albicans

Broth microdilution was used to determine the antifungal activity of synthetic dialk(en)ylthiosulfinates and the corresponding TGS against *C. albicans*, revealing MIC values within 0.69–3.31 μg/mL and 0.36–1.1 μg/mL, respectively (Table 3).

Recent studies have shown that the antimicrobial activity of dialk(en)ylthiosulfinates might be associated with their ability to penetrate the walls of microbial cells [9,18]. Unlike S-alk(en)yl-L-cysteine sulfoxides, dialk(en)ylthiosulfinates have lipophilic properties. The permeability coefficient (P) increases in the series DMTS < DETS < DATS < DPTS (logP: −0.21 < 0.64 < 1.35 < 1.61) [9], which is in accordance with our MIC data for synthetic dialkylthiosulfinates and for dialk(en)ylthiosulfinates obtained using the TGS (Table 3). In our case, an increase in the antifungal activity of dialkylthiosulfinates was observed with an extension of the alkyl chain length in their structure. The same tendency was previously found for some Gram-positive and Gram-negative bacteria, yeast and eukaryotic malignant cells in the study of the anticancer activity of dialkylthiosulfinates [9,19]. The only previously known anticandidal thiosulfinate—DATS (allicin)—confirmed its MIC range within the values reported earlier, both for the chemically synthesized form and when extracted from the crushed cloves of *Allium sativum* (MICs against *Candida* species were 1.57–128 μg/mL) [20,21]. Based on the ^1^H NMR data, in 24 h, allicin forms breakdown products, which are predominant in the spectrum, and it seems impossible to accurately determine the MIC for allicin. This fact is evidenced by the large spread of MIC values in the literature. The MIC values of thiosulfinates produced using the TGS turned out to be 2 times smaller than synthetic ones. Evidently, when the antimicrobial thiosulfinates are synthesized directly at the moment of their application and immediately react with free SH groups of cell proteins, the formation of non-active decomposition products is minimized. In addition, the MIC values for DATS and DMTS are very high, i.e., of the same order with those of commercial antifungal drugs (Table 3). Thus, dialk(en)ylthiosulfinates obtained using the TGS have a pronounced advantage over their synthetic analogues as antimycotics.

### 2.3. Combinatorial Effect of Synthetic Dialk(en)ylthiosulfinates and TGS with Known Therapeutic Drugs

The gold standard in the treatment of infections caused by *Candida* spp. includes amphotericin B and azoles administered alone or in combination with each other [22,23]. Both drugs are membrane-active antibiotics, which interact with a target on the cell membrane and violate its integrity [24,25]. The ability of allicin to form transient pores in artificial and bio-membranes may enhance the fungicidal effect of the membrane-associated antimycotics [18]. At the same time, it has been shown that AmpB induces ROS generation followed by protein oxidation, DNA and RNA damage or phospholipid peroxidation, leading to plasma membrane disruption [26]. Allicin can enhance the antifungal activity of Cu^2+^ ions induced by ROS production [27]. There are several publications describing the synergistic effect of allicin with AmB in vitro and in vivo, which can be facilitated by the oxidative damage mediated by allicin [21,28]. The combination of allicin with the DNA-targeting antimycotic 5-flucytosine or with fluconazole and ketoconazole caused a different effect on most of the tested *Candida* spp. isolates [28,29].

**Figure 3:**
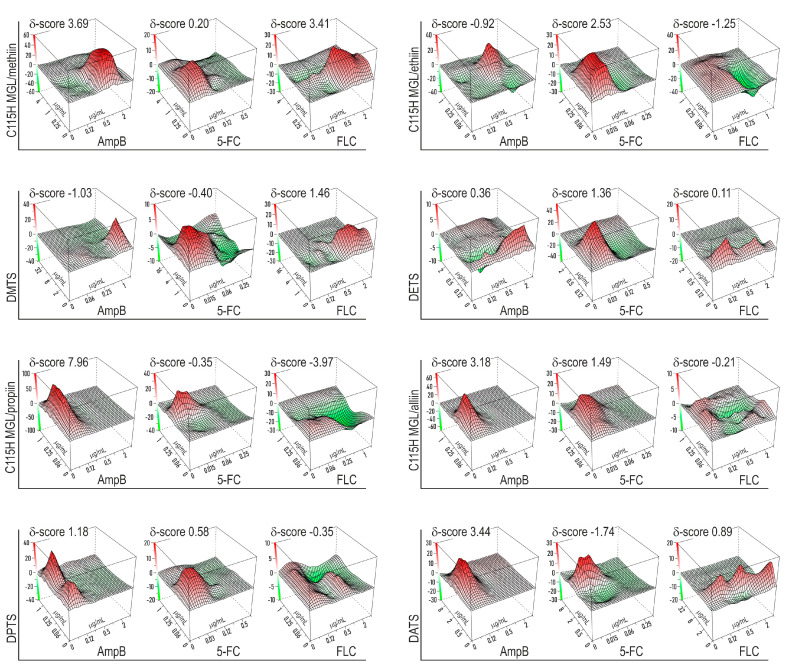
Synergy landscape of combinations of synthetic dialk(en)ylthiosulfinates and TGSs with three common antimicrobial drugs.

**Figure 4:**
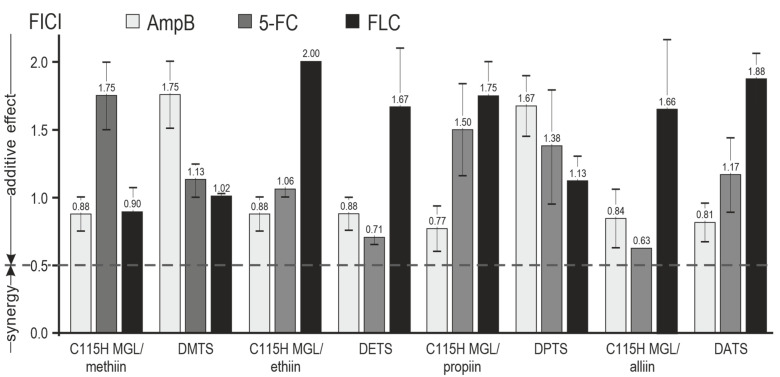
Fractional inhibitory concentration indices (FICIs) of combinations of synthetic thiosulfinates and the TGS with three common antimicrobial drugs.

We have performed a checkerboard assay to evaluate the combinatorial effect of synthetic dialk(en)ylthiosulfinates and the corresponding TGS with commercial antifungal drugs. Data representing the synergy landscape and FICI diagram (Figure 3 and Figure 4) show that combinations of antimycotics with dialk(en)ylthiosulfinates and the TGS enhance the antifungal effect of the commercial drugs in the order FLC < 5-FC < AmpB.

All tested preparations had an additive effect against *C. albicans* in the combination with antimycotics (0.5 < FICI < 4; −10 < ZIP synergy score < 10) (Figure 3 and Figure 4). At certain concentrations, the combinations of thiosulfinates and antimycotics showed pronounced synergism, which is marked with the bright red area on a synergy landscape plot (Figure 3). The effect is compelling, if we consider recent reports on the allicin influence (alone and in combination with AmpB or 5-FC) on the morphology of *C. albicans* cells studied via scanning electron microscopy (SEM) and atomic force microscopy (AFM) techniques [28]. Allicin alone did not cause dramatic damage to fungal cells, whereas the outer membrane of the cells was seriously damaged by the combination of allicin with AmpB. A slightly lower effect was observed for the combination of allicin with 5-FC.

Based on these data, we can conclude that thiosulfinates can penetrate cell membranes and modify both extracellular and intracellular thiol-containing proteins, for example, attack the main glutathione-associated enzymes, exerting an antifungal effect [9]. We suggest that the observed overall additive effect of the tested thiosulfinates in combinations with various antimycotics can be attributed to a wide range of protein targets in the fungal cell.

## 3. Materials and Methods

### 3.1. Materials

Chemicals, solvents, pyridoxal-5′-phosphate (PLP), D,L-dithiothreitol (DTT), alliin, dialk(en)yldisulfides, amphotericin B (AmpB), fluconazole (FLC), 5-flucytosine (5-FC), kanamycin, YPD media and Sabouraud dextrose agar were purchased from Sigma-Aldrich (St. Louis, MO, USA); DEAE-sepharose was from “GE Healthcare” (Chicago, IL, USA); RPMI 1640 media and 3-(N-morpholino)propanesulfonic acid (MOPS) were purchased from PanEco (Moscow, Russia); S-alkyl-L-cysteine sulfoxides (methiin, ethiin and propiin) were synthesized according to the previously reported procedures [13]. 2-Nitro-5-thiobenzoate was obtained as described [30]. *E. coli* strain BL21 (DE3) F-ompT hsdSB gal dcm (DE3) was obtained from Novagen (Darmstadt, Germany). The plasmid with the gene of the *C. freundii* C115H MGL mutant form was obtained previously [13].

### 3.2. Apparatus

NMR spectra were registered on an Avance 3 spectrometer (Bruker BioSpin GmbH, Ettlingen, Germany) with the working frequency of 300 MHz for ^1^H NMR controlled by Topspin v.2.7.

HRMS spectra were registered on a micrOTOF-Q II device (Bruker Daltonics, Billerica, MA, USA); measurements were carried out in positive ion mode; samples were dissolved in acetonitrile and delivered into the mass-spectrometer chamber using an infusion syringe pump.

UV spectra were recorded on a Cary-50 spectrophotometer (Varian, Palo Alto, CA, USA) and an iMark Microplate Reader (Bio-rad, Hercules, CA, USA).

### 3.3. Synthesis of Dialk(en)ylthiosulfinates

Dimethyl-, diethyl-, dipropyl- and diallylthiosulfinates were synthesized based on the previously reported procedures [9]. Briefly, dialk(en)yldisulfide (15 mmol) was dissolved in 5 mL of formic acid and stirred on ice bath for 5 min. Then, 30% H_2_O_2_ (3 mL) was slowly added to the mixture. After 90 min, the reaction was stopped by the addition of 25 mL H_2_O, and the organic compounds were extracted by washing the mixture 2 times with 10 mL of DCM. The solvent was evaporated under reduced pressure at room temperature, and the residue was dissolved in an ethyl acetate:n-hexane mixture (1:2). The product was purified on a silica gel 60 column with the same mixture used as the mobile phase. The product-containing fractions were combined, and the solvents were removed through rotary evaporation at room temperature. NMR spectra were in accordance with the previously reported data [31].

Dimethylthiosulfinate: ^1^H NMR (D_2_O, 300 MHz): 2.65 (s, 3H), 3.03 (s, 3H). HRMS (ESI) of C_2_H_6_OS_2_, *m/z*: calcd for [M-H]- 108.9776, found: 108.9782.

Diethylthiosulfinate: ^1^H NMR (CDCl_3_, 300 MHz): 1.45 (t, J 7.4 Hz, 3H), 1.48 (t, J 7.3 Hz, 3H), 3.17 (q, J 7.4 Hz, 2H), 3.34 (q, J 7.3 Hz, 2H). HRMS (ESI) of C_4_H_10_OS_2_, *m/z*: calcd for [M-H]- 137.0089, found: 137.0093.

Dipropylthiosulfinate: ^1^H NMR (DMSO-d_6_, 300 MHz): 0.98 (t, J 7.3 Hz, 3H), 1.02 (t, J 7.4 Hz, 3H), 1.66–1.82 (m, 4H), 3.04–3.18 (m, 4H). HRMS (ESI) of C_6_H_14_OS_2_, *m/z*: calcd for [M-H]- 165.0402, found: 165.0408.

Diallylthiosulfinate: ^1^H NMR (CDCl_3_, 300 MHz): 3.67–3.79 (m, 4H), 5.15–5.45 (m, 4H), 5.66–5.89 (m, 2H). HRMS (ESI) of C_6_H_10_OS_2_, *m/z*: calcd for [M-H]- 161.0089, found: 161.0094.

### 3.4. Preparation of the C115H MGL

The isolation and purification of C115H MGL was performed as described previously [13]. The activity of the enzyme was determined in the β-elimination reaction of S-methyl-L-cysteine [13]. The specific activity of homogeneous C115H MGL was estimated as 30 U/mg.

### 3.5. Kinetic Measurement via ^1^H NMR

^1^H NMR spectra were recorded at a temperature of 303 K, using a presaturation pulse sequence with a purge element for water signal suppression [32] The automation (AU) program was developed within Topspin to facilitate the recording of the time series of the spectra, starting acquisition at specified time intervals. Each spectrum was accumulated from 4 scans, preceded by 4 dummy scans.

Samples were prepared in phosphate buffered saline (PBS), pH 7.5, with the addition of 10% D_2_O. The enzyme and a substrate were dissolved separately and mixed immediately before placing 0.5 mL of sample of the resulting solution into the NMR tube. To minimize the dead time, initial solutions, as well as the NMR tube, were kept in the thermostat heater prior to mixing. Each sample contained 41 μg of the enzyme and 1.25 mg of the substrate. Signals assigned to pyruvate and thiosulfinate protons were integrated using a modified version of the multi_integ AU program within Topspin. The pyruvate CH_3_ singlet at 2.33 ppm was integrated to determine the kinetic parameters, since it is located in the spectral region clear of any other interfering signals, which makes it easy to integrate. Substrate and product concentrations were derived from NMR integral intensities; 4.76 mM pyruvate solution registered under the same experimental conditions as the spectra of reaction mixtures was used as an external reference standard for quantification. Initial turnover rates were determined based on linear fits of the observed pyruvate concentration relative to the concentration of MGL active units, starting from the initial 10 min (30 min in the case of DMTS) of the monitored reaction. Turnover rates at half-conversion were determined in a similar way as the slope of the kinetic curve at the half-conversion time. The latter was defined as the time interval after which half of the final pyruvate concentration was achieved. Reaction slowdown rates were determined as a relative decrease in the turnover rate after the half-conversion time.

Of note, the resulting integral intensities of the signals belonging to different chemical moieties were not accurately proportional to the number of nuclei, as they were skewed by the NOE and/or saturation effects due to water signal suppression. Plots of the resulting time series data and least-square fits were produced using the Gnuplot program [33].

### 3.6. Determination of Enzymatically Generated Dialk(en)ylthiosulfinates

The solutions of S-(allyl/alkyl)-L-cysteine sulfoxides (10 mg/mL) in the 50 mM potassium phosphate buffer, pH 7.5, with 0.1 mM PLP were combined with C115H MGL (0.14 U/mL) in the same buffer and incubated for 1 h at room temperature. The concentration of thiosulfinates formed in the reaction mixtures was determined using an NTB assay [34].

### 3.7. Cell Culture

The *Candida albicans* ATCC 10231 strain was obtained from the American Type Culture Collection (ATCC; Rockville, MD, USA). Subculturing and inoculum preparation were carried out according to Clinical and Laboratory Standards Institute (CLSI) recommended method M27-A3, with an incubation temperature of 35 °C [35]. The yeast stock suspension of 1 × 10^6^ to 5 × 10^6^ cells per mL was prepared in sterile saline. A working suspension of 5.0 × 10^2^ to 2.5 × 10^3^ cells per mL was prepared via two-step dilution of the stock suspension with RPMI 1640 broth medium.

### 3.8. Antifungal Activity Evaluation

The minimal inhibitory concentration (MIC) was determined through the broth microdilution (two-fold serial dilutions) method, as described in the CLSI M27-A3, at least five times in triplicate [35]. In accordance with M27-A3, stock solutions of 5-FC and FCL were prepared in sterile water at a concentration of 1280 μg/mL for both. The AmpB stock solution was prepared in DMSO at a concentration of 6400 μg/mL. Stock solutions of thiosulfinates except DATS were prepared in sterile water at a concentration of 1280 μg/mL. The DATS stock solution was prepared in DMSO at a concentration of 6400 μg/mL. Antifungal stock solutions were diluted to a final strength in the test medium and dispensed into the wells of Rows 1 to 10 of the microdilution plates in 100 μL volumes. Then, 100 μL of the inoculum was added to each well. Rows 11 and 12 were used for positive (only inoculums) and negative (only RPMI media) controls. Final tested antifungal concentration ranges were as follows: AmpB, 0.0313 to 16 μg/mL; 5-FC, 0.125 to 64 μg/mL; FLC, 0.125 to 64 μg/mL; thiosulfinates, 0.0625 to 32 μg/mL. MIC endpoints were registered after a 24 h incubation using a microplate absorbance reader. MIC values were determined as the lowest concentration of drug that caused significant (~80%) (fluconazole, 5-flucytosine) or complete (all other agents) growth diminution levels compared to the growth control.

### 3.9. Checkerboard Assay

Checkerboard synergy testing was performed in triplicate with 96-well plates using an 8-by-8 well configuration [36] The freshly prepared stock solutions and serial two-fold dilutions of the testing preparations and commercial antimycotics were prepared as described above. Each well of the 96-well plate contained the tested substance on one axis in a combination with the commercial antimycotic along the other axis in an equal volume of 50 μL. The concentrations were ranged from 4× MIC to 1/16× MIC and included a zero concentration for each antifungal agent. Then, 100 μL of inoculum was added to each well, and the plate was incubated at 35 °C for 24 h. In the case of AmpB and DATS, the total DMSO concentration in a well did not exceed 1%. Results were registered using a microplate absorbance reader.

The interpretation of checkerboard synergy test results was performed by calculating of the Fractional Coefficient of Inhibition Index (FICI) [36]. The fractional inhibitory concentration (FIC) was calculated as follows: FICA = MIC of drug A in combination/MIC of drug A alone. The fractional inhibitory coefficient index (FICI) values were calculated as the FICA + FICB, both from the same well. FICI values were interpreted as synergistic if FICI ≤ 0.5, additive if 0.5 < FICI ≤ 4 and antagonistic if FICI > 4.0. The ZIP synergy score was determined using the SynergyFinder web application [37,38].

### 3.10. Statistical Analysis

The results were reported as the mean ± standard deviation (SD) or mean ± 95% confidence interval CI (95%) using the MS Excel Statistical Analysis tool pack. Drug combination landscapes were visualized using the SynergyFinder web application server [38].

## 4. Conclusions

The two-component system for the treatment of *Candida* infections allows for therapeutically relevant concentrations of antimycotic thiosulfinates to be obtained at the desired localized area under release-controllable conditions. Such treatment can lower the overall toxic effects and potentiate the therapeutic efficacy of the treatment. For the first time, the ^1^H NMR method was used to show that the TGS (methionine γ-lyase + S-alk(en)yl-L-cysteine sulfoxides binary system) effectively generates antifungal thiosulfinates. TGS appeared to have better anticandidal efficacy in comparison with synthetic dialk(en)ylthiosulfinates. Thiosulfinates, both synthetic and in situ TGS-originated, revealed an additive effect when combined with commercial antifungals. The observed effect can be explained by the presence of multiple targets for the action of the thiosulfinates in the pathogen cell, which may diminish the emergence of pathogen resistance for a prolonged time and may be utilized for surface antimycotic treatment on medically implanted devices.

## Figures and Tables

**Table 1:** ^1^H NMR signals of enzymatically generated thiosulfinates found in kinetic measurements.

DMTS	
low field	CH_3_: 3.04 (s)
high field	CH_3_: 2.66 (s)
DETS	
low field	CH_3_: 1.40 (t, 7.4 Hz)
high field	CH_3_: 1.33 (t)
other signals	3.27 (dq), 3.19 (dq), 3.18 (q)
DPTS	
low field	CH_3_: 1.02 (t, 7.4 Hz)
high field	CH_3_: 0.97 (t)
other signals	3.12–3.24 (m), 1.68–1.85 (m)
DATS	
low field	=CH_2_ (cis): 5.49 (d ^1^, 10.1 Hz)=CH_2_ (trans): 5.46 (d ^1^, 16.9 Hz)
high field	=CH_2_ (cis): 5.22 (d ^1^)=CH_2_ (trans): 5.32 (d ^1^)
other signals	5.80–6.04 (m), 3.74–4.01 ^2^ (m)
Pyruvate	2.33 (s)

^1^ Poorly resolved ^2^*J* and ^4^*J* are observed (ddd, <2 Hz). ^2^ A strong overlap with the corresponding substrate signal.

**Table 2:** Kinetic parameters of enzymatic thiosulfinate production.

	DMTS	DETS	DPTS	DATS
Initial turnover rate ^1^, s^−1^	0.23 ± 0.01	1.03 ± 0.09	0.97 ± 0.04	1.56 ± 0.04
Turnover rate at half-conversion, s^−1^	0.07 ± 0.002	0.39 ± 0.01	0.39 ± 0.01	0.75 ± 0.08
Half-conversion time, min	231	29	29	14
Reaction slowdown rate ^2^	70%	62%	60%	52%

^1^ The concentration of MGL active sites was 1.95 μM. ^2^ Relative decrease in the turnover rate after reaching a half-conversion time.

**Table 3:** MIC values of synthetic thiosulfinates, TGS and commercial drugs against *C. albicans*.

Antimycotic Drug	*C. albicans,* MIC ± CI (95%)	
	μg/mL	μM
Synthetic thiosulfinates		
DMTS	2.68 ± 0.57	24 ± 5.1
DETS	0.72 ± 0.16	5.2 ± 1.15
DPTS	0.69 ± 0.26	4.1 ± 1.54
DATS	3.31 ± 1.10	20 ± 6.65
TGSs		
C115H MGL/methiin	1.11 ± 0.22	10 ± 1.98
C115H MGL/ethiin	0.41 ± 0.09	3 ± 0.66
C115H MGL/propiin	0.36 ± 0.09	2.2 ± 0.55
C115H MGL/alliin	0.39 ± 0.07	2.4 ± 0.43
Commercial drugs		
AmpB	0.46 ± 0.25	0.5 ± 0.27
FLC	1.06 ± 0.21	3.5 ± 0.69
5-FC	0.16 ± 0.03	1.2 ± 0.22

## Data Availability

Data is contained within the article and Supplementary Material.

## Supplementary Materials

The following supporting information can be downloaded at: https://www.mdpi.com/article/10.3390/ph16121695/s1, Figure S1: ^1^H NMR spectrum of dimethylthiosulfinate; Figure S2: ^1^H NMR spectrum of diethylthiosulfinate; Figure S3: ^1^H NMR spectrum of dipropylthiosulfinate; Figure S4: ^1^H NMR spectrum of products of the enzymatic formation of dimethylthiosulfinate; Figure S5: ^1^H NMR spectrum of products of the enzymatic formation of diethylthiosulfinate; Figure S6: ^1^H NMR spectrum of products of the enzymatic formation of dipropylthiosulfinate; Figure S7: ^1^H NMR spectrum of products of the enzymatic formation of diallylthiosulfinate; Figure S8: Full kinetic data plots based on the integration of NMR signals; Figure S9: Representative examples of MIC determination experiments in 96-well plates (spectrophotometric data). (a) Plate with C115H MGL/alliin and DATS; (b) plate with C115H MGL/methiin and DMTS; (c) plate with C115H MGL/ethiin and DETS; (d) plate with C115H MGL/propiin and DPTS; (e) plate with C115H MGL/propiin and AmpB; (f) plate with 5-FC; (g) plate with FLC.
