# Supplementary material for: Anticandidal Activity of In Situ Methionine γ-Lyase-Based Thiosulfinate Generation System vs. Synthetic Thiosulfinates
Source: Pharmaceuticals (Basel). 2023 Dec 7;16(12):1695. doi: 10.3390/ph16121695 (PMC10748059; doi:10.3390/ph16121695)
Supplement: Supplementary file 1 [file pharmaceuticals-16-01695-s001.zip › pharmaceuticals-2695340-supplementary.pdf]

ELECTRONIC SUPPLEMENTARY MATERIAL

Anticandidal activity of *in situ* methionine γ-lyase-based thiosulfinates generation system vs. synthetic thiosulfinates

Svetlana Revtovich,[a] Anna Lyfenko,[a] Yaroslav Tkachev,[a] Vitalia Kulikova,[a] Vasiliy Koval,[a] Vladimir Puchkov,[a] Natalya Anufrieva,[a] Pavel Solyev*[a] and Elena Morozova*[a]

[a] *Engelhardt Institute of Molecular Biology of the Russian Academy of Sciences, 32 Vavilov St., 119991 Moscow, Russia*
**Correspondence address: elmorozova@yahoo.com, solyev@gmail.com*

Figure S1. ^{1}H NMR spectrum of dimethylthiosulfinate (D_2O) ---------------------------------- S2
Figure S2. ^{1}H NMR spectrum of diethylthiosulfinate ($CDCl_3$) ---------------------------------- S3
Figure S3. ^{1}H NMR spectrum of dipropylthiosulfinate (DMSO-d_6) ----------------------------- S4
Figure S4. ^{1}H NMR spectrum of products of the enzymatic formation of dimethylthiosulfinate (aqueous PBS buffer pH 7.5 + 10% D_2O)------------------- S5
Figure S5. ^{1}H NMR spectrum of products of the enzymatic formation of diethylthiosulfinate (aqueous PBS buffer pH 7.5 + 10% D_2O) --------------------- S6
Figure S6. ^{1}H NMR spectrum of products of the enzymatic formation of dipropylthiosulfinate (aqueous PBS buffer pH 7.5 + 10% D_2O) ------------------- S7
Figure S7. ^{1}H NMR spectrum of products of the enzymatic formation of diallylthiosulfinate (aqueous PBS buffer pH 7.5 + 10% D_2O)---------------------- S8
Figure S8. Full kinetic data plots based on the integration of NMR signals -------------------- S9
Figure S9. Representative examples of MIC determination experiments in 96-well plates (spectrophotometric data). a) Plate with C115H MGL/alliin and DATS; b) plate with C115H MGL/methiin and DMTS; c) plate with C115H MGL/ethiin and DETS; d) plate with C115H MGL/propiin and DPTS; e) plate with C115H MGL/propiin and AmpB; f) plate with 5-FC; g) plate with FLC. --- S10

Dimethylthiosulfinate (D2O, 300 Mhz)

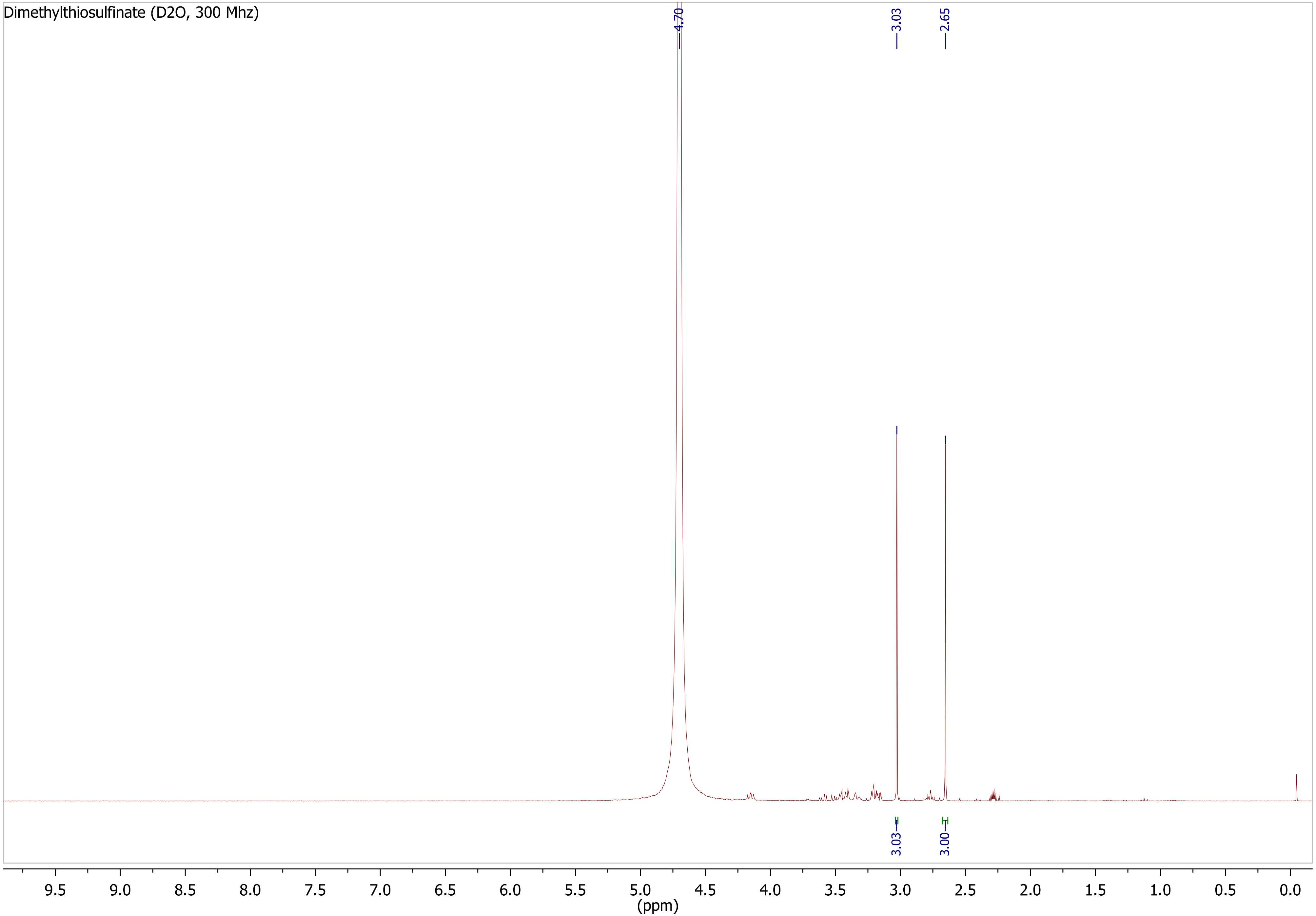

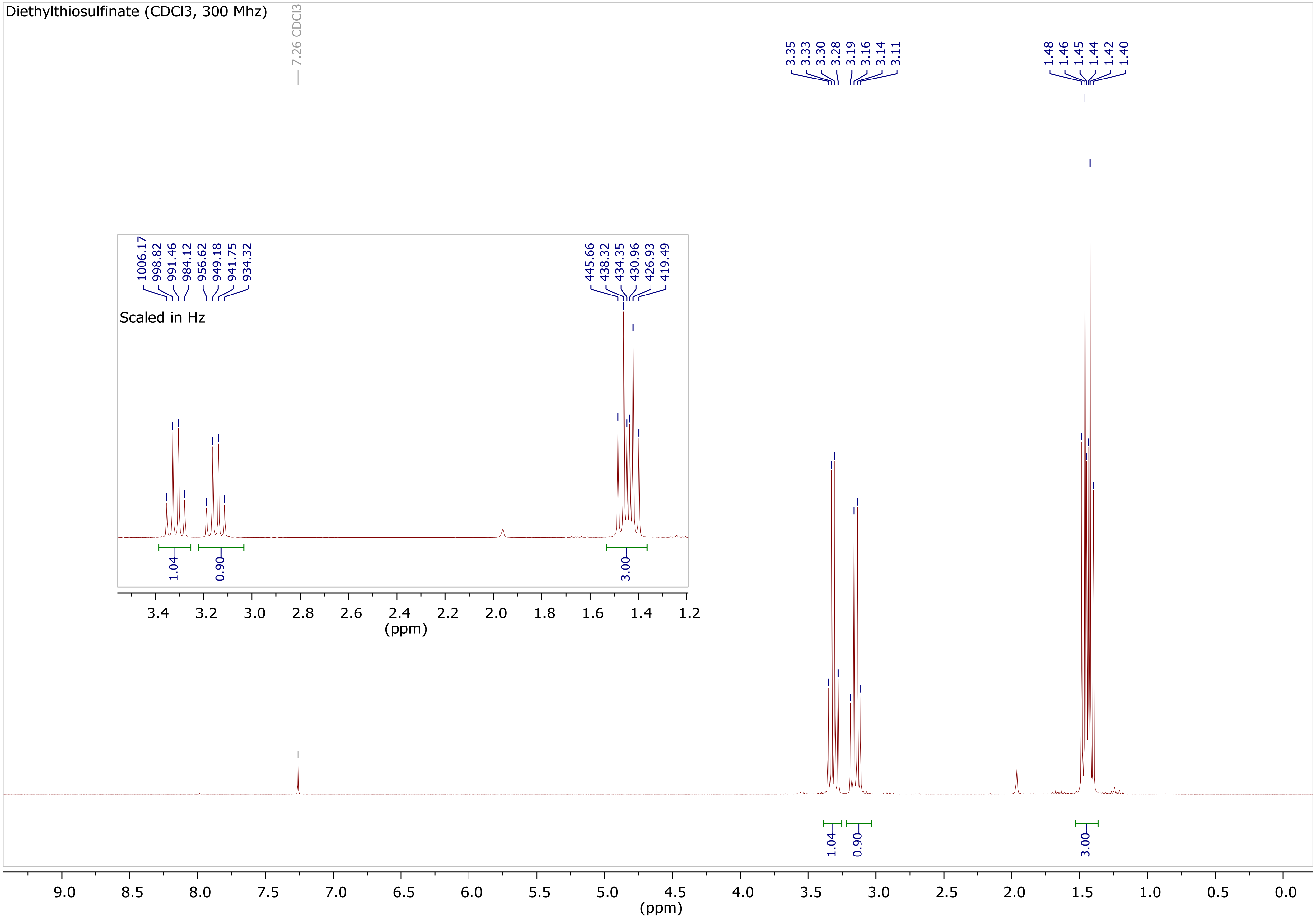

Diethylthiosulfinate (CDCl3, 300 Mhz)
7.26 CDCl3
3.35
3.33
3.30
3.28
3.19
3.16
3.14
3.11
1.48
1.46
1.45
1.44
1.42
1.40
1006.17
998.82
991.46
984.12
956.62
949.18
941.75
934.32
445.66
438.32
434.35
430.96
426.93
419.49
Scaled in Hz
1.04
0.90
3.00
(ppm)

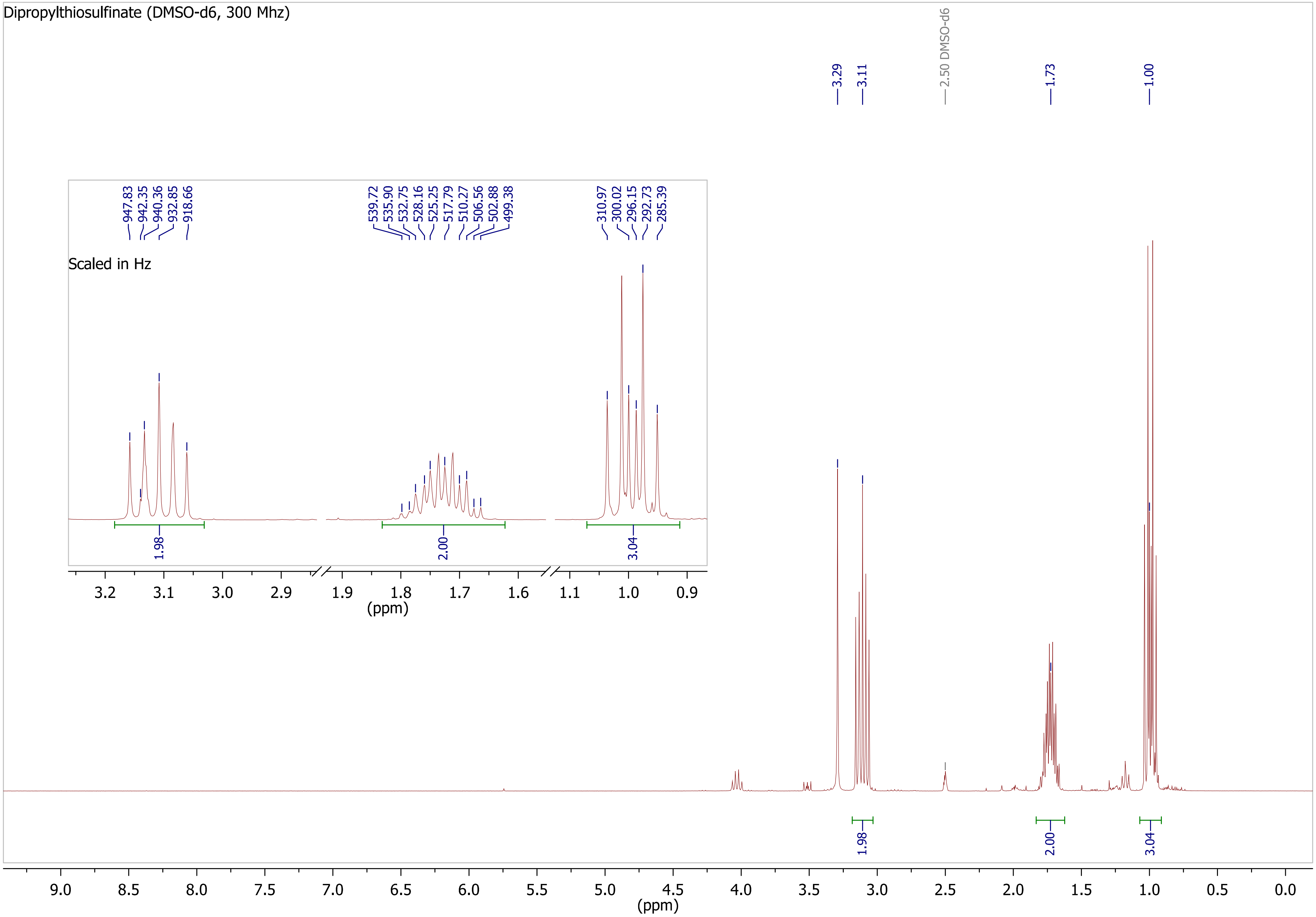
Dipropylthiosulfinate (DMSO-d6, 300 Mhz)
3.29
3.11
2.50 DMSO-d6
1.73
1.00
947.83
942.35
940.36
932.85
918.66
539.72
535.90
532.75
528.16
525.25
517.79
510.27
506.56
502.88
499.38
310.97
300.02
296.15
292.73
285.39
Scaled in Hz
1.98
2.00
3.04
(ppm)

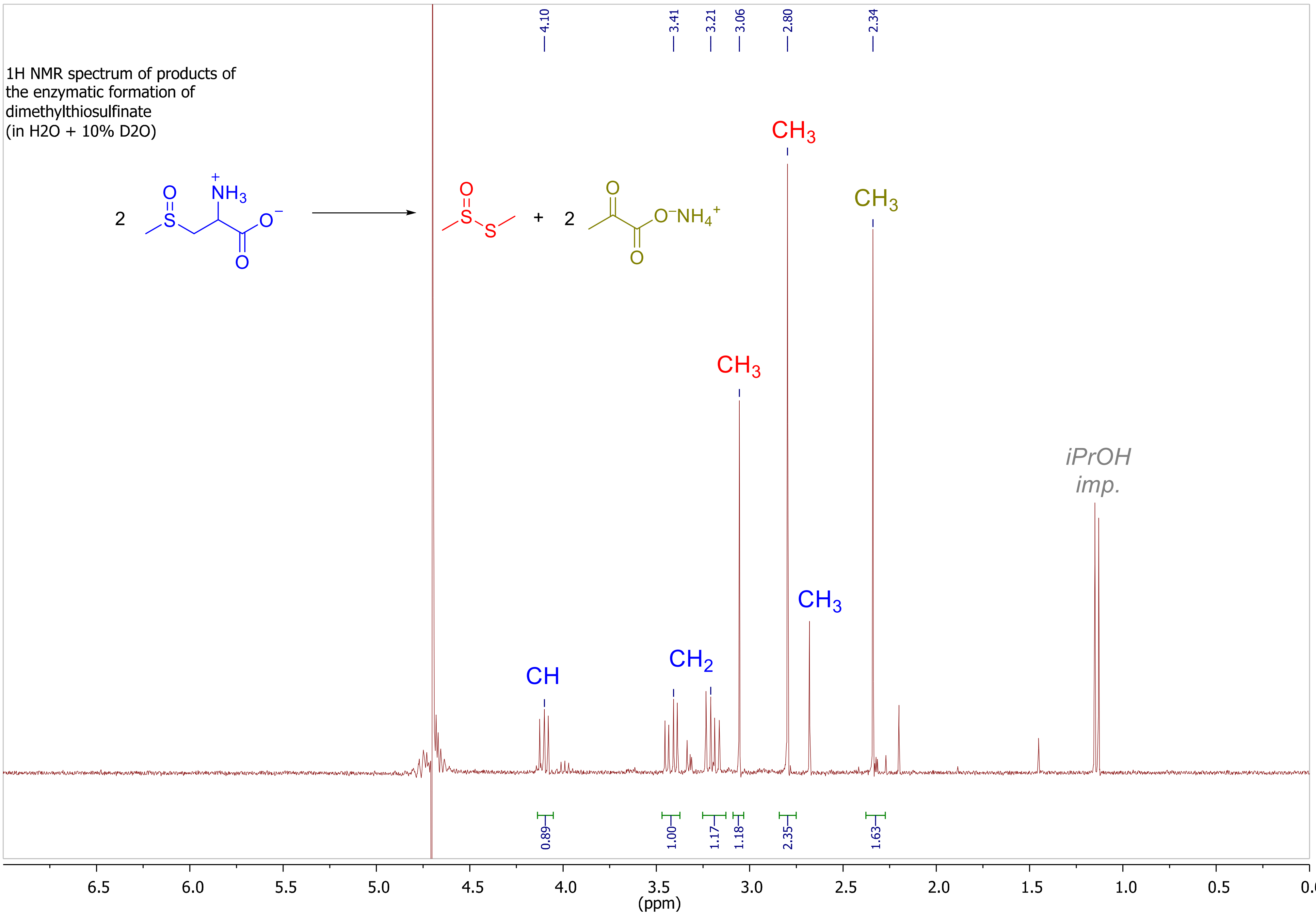
1H NMR spectrum of products of
the enzymatic formation of
dimethylthiosulfinate
(in H2O + 10% D2O)
2
O
NH3
O
O
S
+
2
O
S
S
O
O
O NH4
CH3
CH3
CH3
CH3
CH
CH2
iPrOH
imp.
4.10
3.41
3.21
3.06
2.80
2.34
0.89
1.00
1.17
1.18
2.35
1.63
6.5
6.0
5.5
5.0
4.5
4.0
3.5
3.0
2.5
2.0
1.5
1.0
0.5
0.0
(ppm)

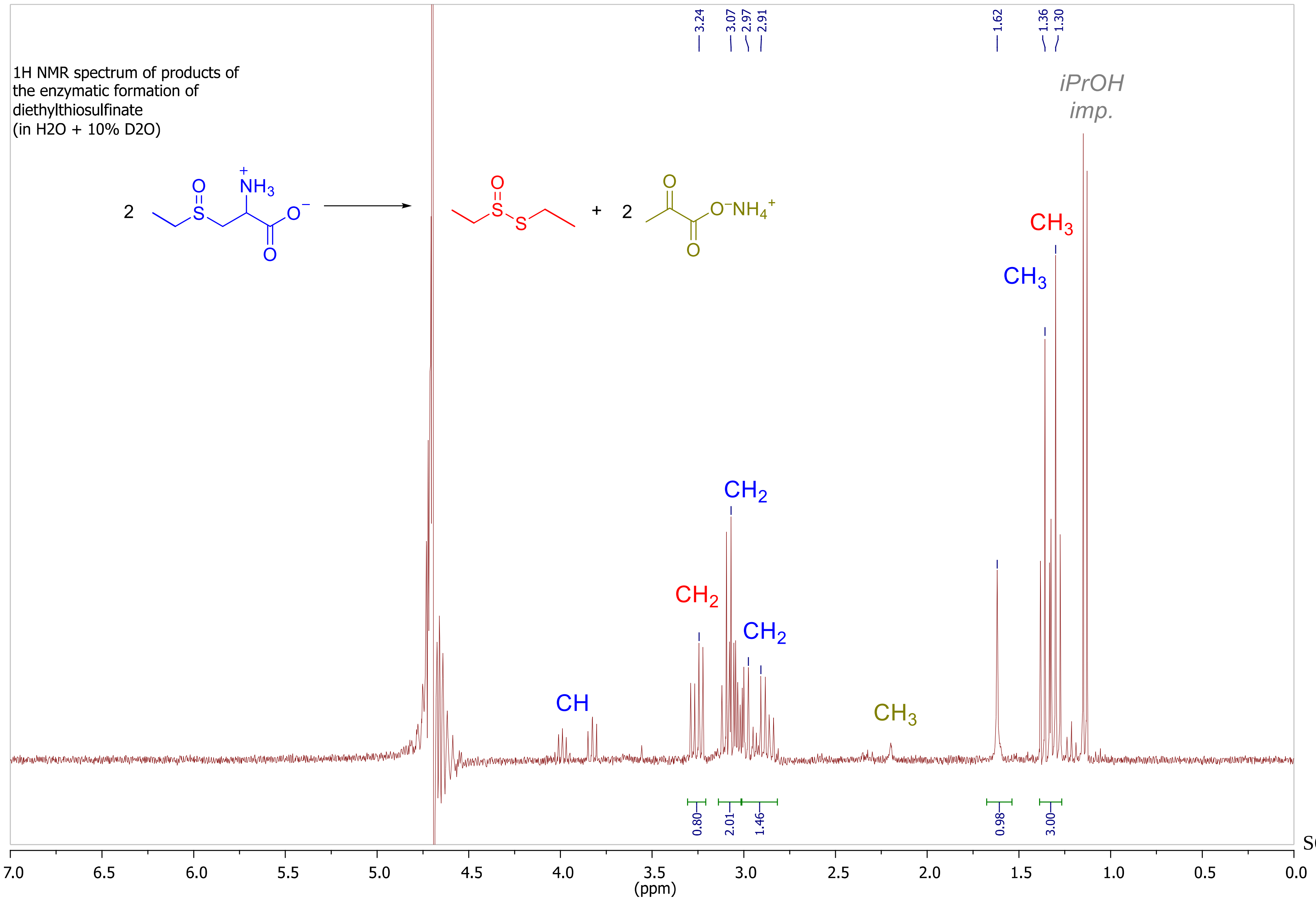
1H NMR spectrum of products of the enzymatic formation of diethylthiosulfinate (in H2O + 10% D2O)
2
NH3
O
S
O
+
2
O
S
S
2
O
O⁻NH4⁺
O
3.24
3.07
2.97
2.91
1.62
1.36
1.30
iPrOH imp.
CH3
CH3
CH2
CH2
CH2
CH
CH3
0.80
2.01
1.46
0.98
3.00
7.0
6.5
6.0
5.5
5.0
4.5
4.0
3.5
3.0
2.5
2.0
1.5
1.0
0.5
0.0
(ppm)

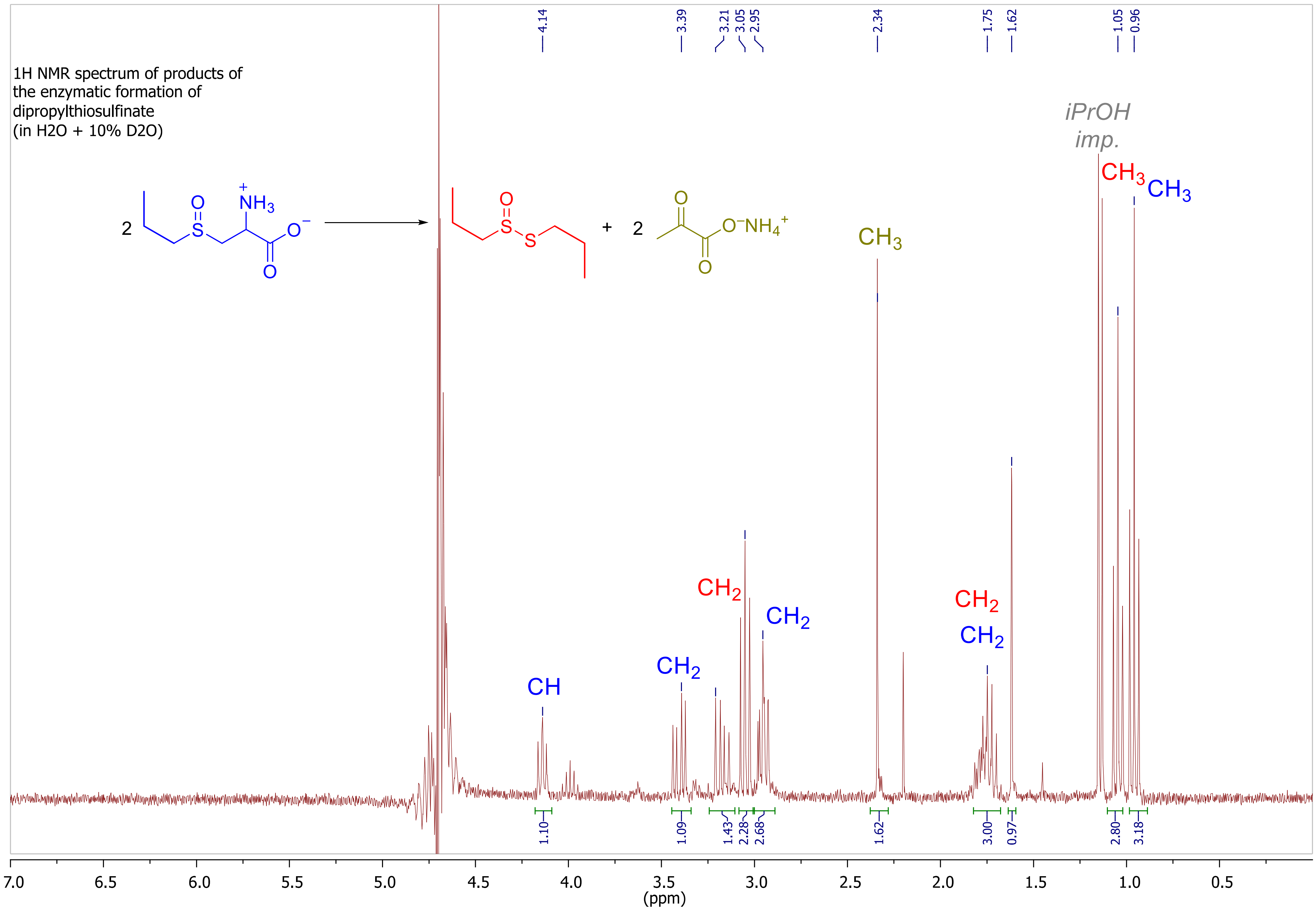
1H NMR spectrum of products of
the enzymatic formation of
dipropylthiosulfinate
(in H2O + 10% D2O)
4.14
3.39
3.21
3.05
2.95
2.34
1.75
1.62
1.05
0.96
iPrOH
imp.
CH3
CH3
CH3
CH2
CH2
CH2
CH2
CH2
CH
1.10
1.09
1.43
2.28
2.68
1.62
3.00
0.97
2.80
3.18
7.0
6.5
6.0
5.5
5.0
4.5
4.0
3.5
3.0
2.5
2.0
1.5
1.0
0.5
(ppm)

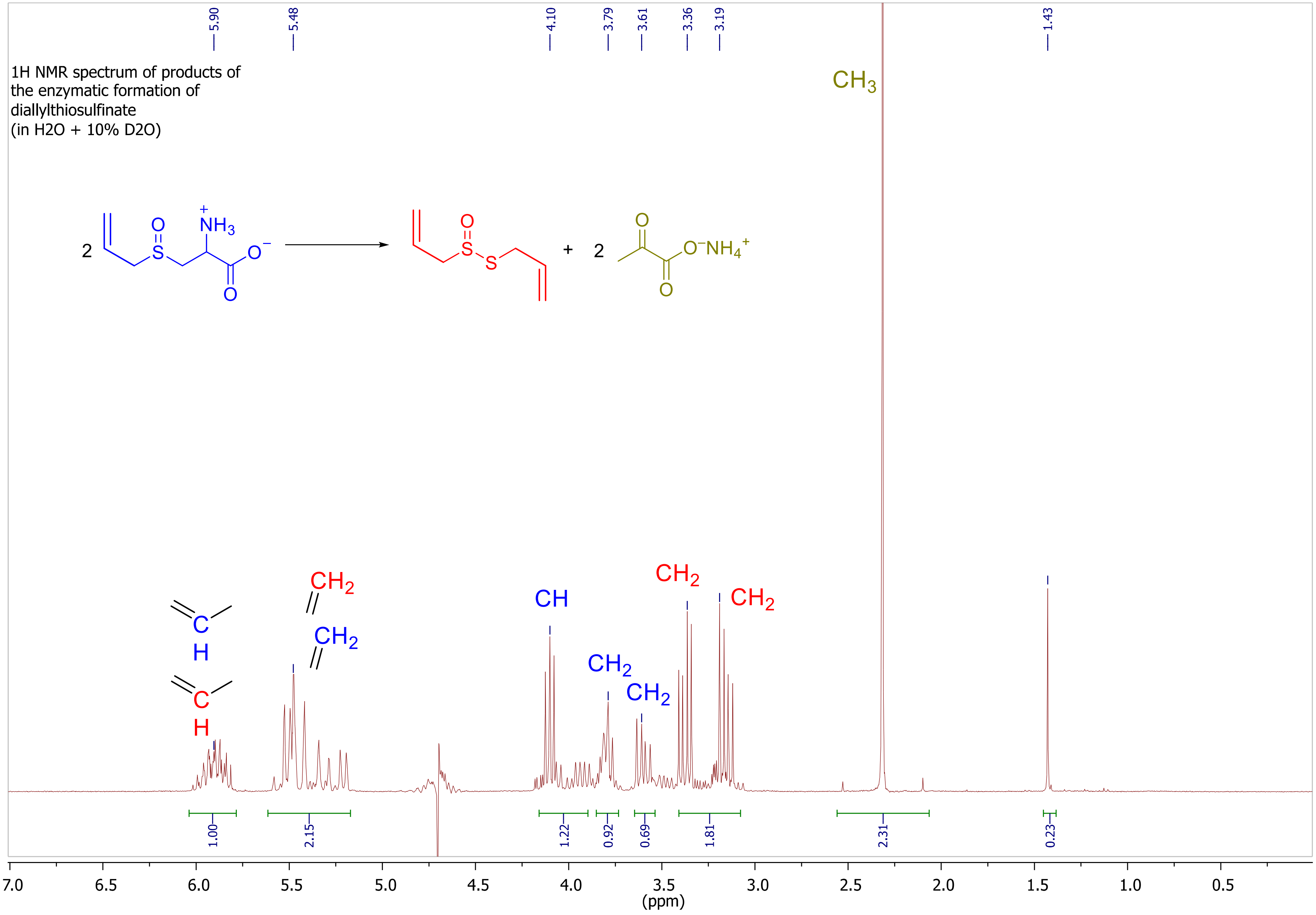

1H NMR spectrum of products of the enzymatic formation of diallylthiosulfinate (in H2O + 10% D2O)
5.90
5.48
4.10
3.79
3.61
3.36
3.19
1.43
CH3
2
NH3
O
S
O
+
2
O
O-NH4+
CH
CH2
1.00
2.15
1.22
0.92
0.69
1.81
2.31
0.23
(ppm)

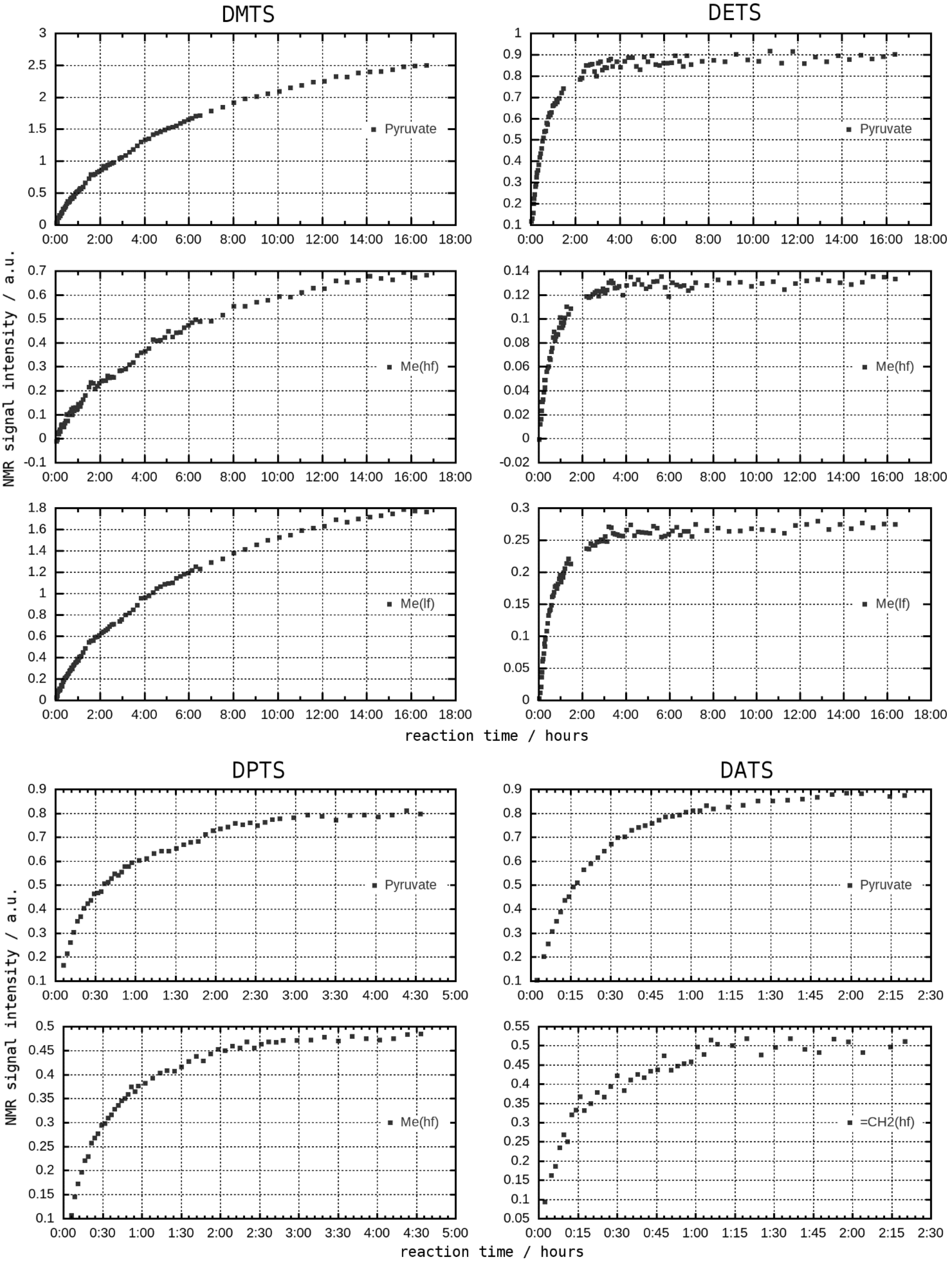

Figure S8. Full kinetic data plots of enzymatic production of thiosulfinates measured by ^{1}H NMR. Abbreviations: Me(hf), high field terminal methyl group signals in DMTS, DETS and DPTS; Me(lf), the same for low field signals; $=CH_2$(hf), high field terminal methylene group of allyl moieties in DATS

Figure S9. Representative examples of MIC determination experiments in 96-well plates (spectrophotometric data). a) Plate with C115H MGL/alliin and DATS; b) plate with C115H MGL/methiin and DMTS; c) plate with C115H MGL/ethiin and DETS; d) plate with C115H MGL/propiin and DPTS; e) plate with C115H MGL/propiin and AmpB; f) plate with 5-FC; g) plate with FLC. MIC values are shown in yellow, growth areas are shown in gray. In the case of testing substances with different concentration ranges (e, f), the concentration range for the first substance (lines B,C,D) is indicated at the top in black, for the second (lines F,G,H) it is indicated at the bottom in brown.

	µg/mL	32	16	8	4	2	1	0.5	0.25	0.125	0.062		
		1	2	3	4	5	6	7	8	9	10	11	12
	A	0.057	0.057	0.051	0.045	0.055	0.056	0.05	0.045	0.041	0.048	0.246	0.055
C115H MGL/ alliin	B	0.053	0.049	0.048	0.045	0.046	0.048	0.05	0.145	0.262	0.281	0.331	0.038
	C	0.051	0.045	0.043	0.042	0.041	0.043	0.046	0.098	0.218	0.28	0.299	0.039
	D	0.055	0.047	0.047	0.045	0.046	0.046	0.05	0.171	0.229	0.268	0.296	0.041
DATS	E	0.048	0.047	0.047	0.073	0.169	0.233	0.262	0.269	0.307	0.285	0.281	0.043
	F	0.05	0.048	0.048	0.087	0.176	0.23	0.273	0.271	0.263	0.277	0.277	0.042
	G	0.043	0.043	0.044	0.066	0.168	0.21	0.263	0.28	0.307	0.298	0.306	0.04
	H	0.047	0.046	0.047	0.045	0.043	0.045	0.042	0.045	0.04	0.053	0.27	0.039
												K+	K-

(a) 96-well plate with C115H MGL/alliin and DATS

	µg/mL	32	16	8	4	2	1	0.5	0.25	0.125	0.062		
		1	2	3	4	5	6	7	8	9	10	11	12
	A	0.055	0.056	0.053	0.048	0.056	0.058	0.051	0.053	0.055	0.052	0.281	0.037
C115H MGL/ methiin	B	0.051	0.055	0.052	0.045	0.047	0.1	0.247	0.27	0.287	0.294	0.277	0.038
	C	0.054	0.051	0.058	0.052	0.051	0.092	0.218	0.272	0.254	0.277	0.265	0.041
	D	0.058	0.052	0.058	0.056	0.063	0.103	0.265	0.273	0.292	0.287	0.285	0.052
DMTS	E	0.048	0.049	0.05	0.051	0.116	0.229	0.279	0.272	0.273	0.279	0.265	0.043
	F	0.049	0.046	0.049	0.05	0.115	0.186	0.255	0.28	0.265	0.283	0.253	0.042
	G	0.047	0.046	0.046	0.048	0.106	0.233	0.27	0.279	0.263	0.275	0.286	0.037
	H	0.044	0.045	0.046	0.053	0.05	0.054	0.052	0.052	0.055	0.056	0.317	0.039
												K+	K-

(b) 96-well plate with C115H MGL/methiin and DMTS

	µg/mL	32	16	8	4	2	1	0.5	0.25	0.125	0.062		
		1	2	3	4	5	6	7	8	9	10	11	12
	A	0.052	0.048	0.05	0.052	0.042	0.056	0.052	0.049	0.049	0.047	0.297	0.053
C115H MGL/ ethiin	B	0.051	0.048	0.06	0.049	0.046	0.045	0.059	0.219	0.29	0.295	0.288	0.04
	C	0.055	0.049	0.063	0.053	0.048	0.046	0.064	0.189	0.254	0.275	0.309	0.044
	D	0.046	0.048	0.062	0.054	0.051	0.052	0.053	0.194	0.274	0.298	0.295	0.042
DETS	E	0.047	0.047	0.047	0.045	0.046	0.055	0.185	0.298	0.288	0.269	0.27	0.042
	F	0.041	0.041	0.041	0.042	0.04	0.048	0.165	0.269	0.279	0.304	0.277	0.038
	G	0.044	0.044	0.046	0.045	0.045	0.053	0.187	0.244	0.283	0.262	0.277	0.041
	H	0.047	0.047	0.048	0.047	0.047	0.057	0.041	0.045	0.045	0.046	0.306	0.043
												K+	K-

(c) 96-well plate with C115H MGL/ethiin and DETS

		µg/mL	32	16	8	4	2	1	0.5	0.25	0.125	0.062		
			1	2	3	4	5	6	7	8	9	10	11	12
	A		0.057	0.051	0.046	0.045	0.045	0.046	0.055	0.049	0.046	0.047	0.268	0.039
C115H MGL/ propiin	B		0.057	0.047	0.043	0.042	0.041	0.044	0.046	0.061	0.215	0.241	0.298	0.039
	C		0.06	0.059	0.047	0.046	0.045	0.047	0.048	0.071	0.202	0.232	0.298	0.043
	D		0.055	0.06	0.054	0.065	0.052	0.056	0.063	0.062	0.206	0.254	0.328	0.048
DPTS	E		0.048	0.05	0.047	0.046	0.045	0.046	0.05	0.137	0.221	0.272	0.289	0.042
	F		0.048	0.05	0.048	0.048	0.047	0.047	0.049	0.095	0.26	0.288	0.294	0.042
	G		0.046	0.045	0.046	0.045	0.046	0.043	0.05	0.107	0.273	0.263	0.28	0.039
	H		0.041	0.042	0.04	0.04	0.04	0.041	0.046	0.046	0.047	0.049	0.301	0.049
													K+	K-

(d) 96-well plate with C115H MGL/propiin and DPTS

		µg/mL	32	16	8	4	2	1	0.5	0.25	0.125	0.062		
			1	2	3	4	5	6	7	8	9	10	11	12
	A		0.047	0.043	0.046	0.048	0.048	0.054	0.057	0.043	0.049	0.049	0.042	0.05
C115H MGL/ propiin	B		0.046	0.051	0.058	0.055	0.053	0.048	0.044	0.047	0.076	0.185	0.282	0.041
	C		0.044	0.057	0.055	0.05	0.048	0.043	0.04	0.046	0.069	0.19	0.259	0.038
	D		0.059	0.055	0.055	0.056	0.06	0.049	0.049	0.05	0.074	0.147	0.283	0.04
AmpB	E		0.04	0.056	0.055	0.051	0.047	0.056	0.266	0.22	0.225	0.231	0.252	0.04
	F		0.046	0.052	0.06	0.057	0.054	0.06	0.232	0.256	0.231	0.254	0.261	0.043
	G		0.048	0.05	0.055	0.054	0.054	0.062	0.246	0.255	0.257	0.245	0.258	0.044
	H		0.043	0.043	0.042	0.042	0.044	0.051	0.046	0.049	0.053	0.057	0.261	0.038
		µg/mL	16	8	4	2	1	0.5	0.25	0.125	0.062	0.031	K+	K-

(e) 96-well plate with C115H MGL/propiin and AmpB

		µg/mL	64	32	16	8	4	2	1	0.5	0.25	0.125		
			1	2	3	4	5	6	7	8	9	10	11	12
	A		0.048	0.05	0.049	0.049	0.05	0.048	0.047	0.044	0.045	0.048	0.29	0.041
5-FC	B		0.048	0.049	0.048	0.045	0.044	0.045	0.042	0.041	0.041	0.049	0.296	0.04
	C		0.058	0.056	0.054	0.053	0.051	0.051	0.047	0.044	0.043	0.051	0.268	0.043
	D		0.061	0.07	0.066	0.07	0.066	0.062	0.063	0.051	0.061	0.066	0.269	0.054
5-FC	E		0.058	0.056	0.053	0.052	0.051	0.049	0.049	0.062	0.105	0.169	0.265	0.046
	F		0.056	0.055	0.053	0.052	0.05	0.049	0.049	0.057	0.099	0.153	0.269	0.044
	G		0.074	0.049	0.048	0.047	0.047	0.046	0.047	0.066	0.119	0.17	0.287	0.042
	H		0.042	0.044	0.044	0.041	0.042	0.043	0.043	0.049	0.04	0.049	0.289	0.039
		µg/mL	16	8	4	2	1	0.5	0.25	0.125	0.062	0.031	K+	K-

(f) 96-well plate with 5-FC

		µg/mL	64	32	16	8	4	2	1	0.5	0.25	0.125		
			1	2	3	4	5	6	7	8	9	10	11	12
	A		0.04	0.038	0.039	0.037	0.036	0.035	0.039	0.032	0.037	0.039	0.318	0.034
FLC	B		0.047	0.047	0.052	0.048	0.06	0.054	0.091	0.245	0.342	0.37	0.369	0.031
	C		0.047	0.047	0.05	0.053	0.057	0.058	0.08	0.218	0.319	0.328	0.321	0.038
	D		0.045	0.045	0.052	0.047	0.055	0.059	0.087	0.209	0.344	0.345	0.349	0.044
	E		0.034	0.034	0.034	0.034	0.034	0.036	0.039	0.035	0.034	0.04	0.337	0.043
	F		0.034	0.034	0.035	0.035	0.035	0.035	0.032	0.032	0.04	0.035	0.342	0.042
	G		0.034	0.035	0.034	0.035	0.035	0.036	0.035	0.034	0.038	0.032	0.338	0.035
	H		0.044	0.045	0.045	0.033	0.038	0.037	0.044	0.038	0.039	0.038	0.334	0.034
													K+	K-

(g) 96-well plate with FLC
